# Interaction between lipoprotein (a) levels and body mass index in first incident acute myocardial infarction

**DOI:** 10.1186/s12872-020-01626-7

**Published:** 2020-07-28

**Authors:** Ruo-Ling Teng, Heng Wang, Bei-Chen Sun, Dong-Ping Cai, Yong-Ming He

**Affiliations:** 1 Division of Cardiology, the First Affiliated Hospital of Soochow University, Suzhou, Jiangsu Province 215006 P.R. China; 2 Healthcare Center for Shishan Street Community of Suzhou New District, Suzhou, Jiangsu Province 215011 P.R. China

**Keywords:** Interaction, Lipoprotein (a), Body mass index, First incident acute myocardial infarction

## Abstract

**Background:**

Possible interaction between Lipoprotein (a) (Lp(a)) and body mass index (BMI) was investigated with regard to the risk of first incident acute myocardial infarction (AMI).

**Methods:**

Cross-sectional study of 1522 cases with initial AMI and 1691 controls without coronary artery disease (CAD) were retrospectively analyzed using logistic regression model. Subjects were categorized based on Lp(a) and BMI and compared with regard to occurrence of AMI by calculating odds ratios (ORs) with 95% confidence intervals (CIs). A potential interaction between Lp(a) and BMI was evaluated by the measures of effect modification on both additive (Relative excess risk due to interaction, RERI) and multiplicative scales.

**Results:**

Compared with reference group (BMI < 24 kg/m^2^ and in the first quintile of Lp(a)), multivariable-adjusted analysis revealed that ORs(95%CI) of AMI were 2.27(1.46–3.52) for higher BMI alone; 1.79(1.11–2.90), 1.65(1.05–2.60), 1.96(1.20–3.20) and 2.34(1.47–3.71) for higher Lp(a) alone across its quintiles; and 2.86(1.85–4.40), 3.30(2.14–5.11), 4.43(2.76–7.09) and 5.98(3.72–9.60) for both higher BMI and higher Lp(a), greater than the sum of the both risks each. Prominent interaction was found between Lp(a) and BMI on additive scale (RERI = 2.45 (0.36–4.54) at the fifth quintile of Lp(a)) but not on multiplicative scale.

**Conclusions:**

This study demonstrates that BMI and Lp(a) levels are important factors affecting the risk of AMI. Significant interaction is found between Lp(a) and BMI in initial AMI on additive scale, indicating that Lp(a) confers greater risk for initial AMI when BMI is elevated. For those whose BMIs are inadequately controlled, Lp(a) lowering may be an option.

**Trial registration:**

This clinical study was not registered in a publicly available registry because this study was a retrospective study first started in 2015. Data are available via the correspondent.

## Background

Owing to the known difficulties and enormous health burden of treatment of CAD, better primary prevention through lifestyle modifications is a major public health priority [1]. Increasing evidence supports the hypothesis that Lp(a) and BMI are independent risk factors for CAD [2–8]. The synergistic effects of Lp(a) and BMI may be greater than the sum of their separate effects. Illustration of interaction between them may help provide more effective interventions in susceptible subgroups [9]. However, few studies have explored the interaction between them. The current study performed a cross-sectional study using logistic regression model to manifest the interactive effect between Lp(a) and BMI, which may be favourable to reduce the risk of first incident AMI from a new angle [10].

## Methods

### The database

Case collection and scientific research system for clinical cardiology (CCSSSCC) database has been described elsewhere [11]. The establishment and use of the database were approved by the Institutional Review Boards of the First Affiliated Hospital and the Soochow University (No. 2016SZYYLL00598). All patient records were anonymised and the Institutional Review Board relinquished the need for informed consent before analysis owing to the retrospective nature of data. This study is in accordance with the outlined principles of the Declaration of Helsinki.

### Patients

Patient selection has been described elsewhere [11]. In short, this retrospective study included patients hospitalized from January 1, 2010 to December 31, 2013. Exclusion criteria were as follows: 1) patients without Lp(a) examinations; 2) repeat hospitalizations; 3) patients with thyroid dysfunctions; 4) patients with liver and/or kidney dysfunctions; 5) patients with any coexistent entities mentioned above; 6) initial ischemic heart disease; 7) prior CAD; 8) non-CAD patients not confirmed by coronary angiography (CAG). For a patient with multiple hospitalizations, data were collected at the time of first admission. The first laboratory results from multiple laboratory tests performed on hospitalized patients were collected. Information on demographic characteristics, lifestyle, risk factors, laboratory tests, lipid profiles and medications were recorded in detail in our previous studies [11, 12].

### Definitions, diagnoses and grouping

Definitions of smoking, drinking status and diagnosis of CAD, initial ischemic heart disease (IHD), prior CAD, primary hypertension (PH), type 2 diabetes mellitus (DM), thyroid dysfunction, liver dysfunction and kidney dysfunction have been described elsewhere in detail [11]. The definition of the first incident AMI was consistent with the third universal MI definition, and there was no clear past history of MI [13]. A total of 1522 first incident AMI cases was diagnosed with chief complaints, cardiac biomarker examinations, electrocardiogram, coronary angiography, echocardiography, and Holter monitoring, respectively or in combination. A total of 1691 non-CAD controls were all confirmed by normal coronary angiograms. BMI was calculated as weight in kilograms divided by height in meters squared, with a BMI < 24 kg/m^2^ was regarded as normal in this study [14].

Patients were categorized on the basis of Lp(a) and BMI. We categorized all subjects into quintiles (Q1 ≤ 34 mg/l, Q2 ≤ 65 mg/l, Q3 ≤ 118 mg/l, Q4 ≤ 246.9 mg/l and Q5 ≤ 2138 mg/l) on the basis of the serum level of Lp(a), and dichotomized all subjects at a cutoff of 24 kg/m^2^ of BMI [15]. Risk of AMI was assessed based on four groups: 1) Reference group: patients within Q1 of Lp(a) and BMI < 24 kg/m^2^; 2) Group with higher Lp(a) alone: patients within Q2-Q5 of Lp(a) and BMI < 24 kg/m^2^; 3) Group with higher BMI alone: patients within Q1 of Lp(a) and BMI ≥24 kg/m^2^; 4) Group with both higher Lp(a) and higher BMI: patients within Q2-Q5 of Lp(a) and BMI ≥24 kg/m^2^.

### Lab measurements

Lab measurements have been described elsewhere [11]. Blood samples were taken after eight hours of fasting on the second day morning of admission. The latex-enhanced immunoturbidimetric diagnostic reagent kits from Sekisui Diagnostic Ltd. have been used to quantify the Lp(a) concentrations and they are insensitive to the isoforms of Lp(a). The assay range is 10–1000 mg/l. To ensure the Lp(a) concentrations were within the security range of the assay and would not mistakenly be considered as a low concentration due to antigen excess, the blood samples with the Lp(a) > 1000 mg/L were routinely diluted 1:4. Lp(a) protein calibrator provided by Sekisui Co. Ltd., in accordance with the IFCC PRM-2, has been used to calibrate the Lp(a) examination results [11]. Other biochemical indexes and lipid profiles were quantitatively determined according to the manufacturer’s instructions. The intra-assay and inter-assay CVs were 2.5 and 3.11%, respectively.

### Statistical methods

All continuous variables involved in this study did not conform to the normal distribution and were represented by median (inter quartile range, IQR). Rank-sum test was used for group comparison. Categorical variables were represented by frequency and percentage and the chi-square test was used for group comparison. Lp(a) levels were divided into quintiles and the first quintile was used as a reference. Unconditional logistic regression was adopted for model fitting. Crude ORs and adjusted ORs (with the adjustment of eleven factors including age, sex, smoking status, drinking status, diabetes mellitus (DM), primary hypertension (PH), high-density lipoprotein cholesterol (HDL), triglycerides (TG), albumin (Alb), serum creatinine (Cr) and low-density lipoprotein-cholesterol (LDL-C)) were reported. The results were presented in the way recommended by International Journal of Epidemiology [10]. Relative excess risk due to interaction (RERI) was used to evaluate additive interaction, which was calculated for binary variables as RERI_IRR_ = IRR_11_ − IRR_10_ – IRR_01_ + 1 [16]. Multiplicative interaction was assessed using the ratio of IRRs: IRR_11_/(IRR_10_ × IRR_01_) [9]. If 95% CI of RERI does not contain 0, then there is additive interaction; if *P* value of product term in logistic model is < 0.05, then there is multiplication interaction [17–19]. Statistical analyses and graphics were performed using STATA 15.0. Two-tailed P<0.05 was considered to be statistically significant.

## Results

**Fig. 1:**
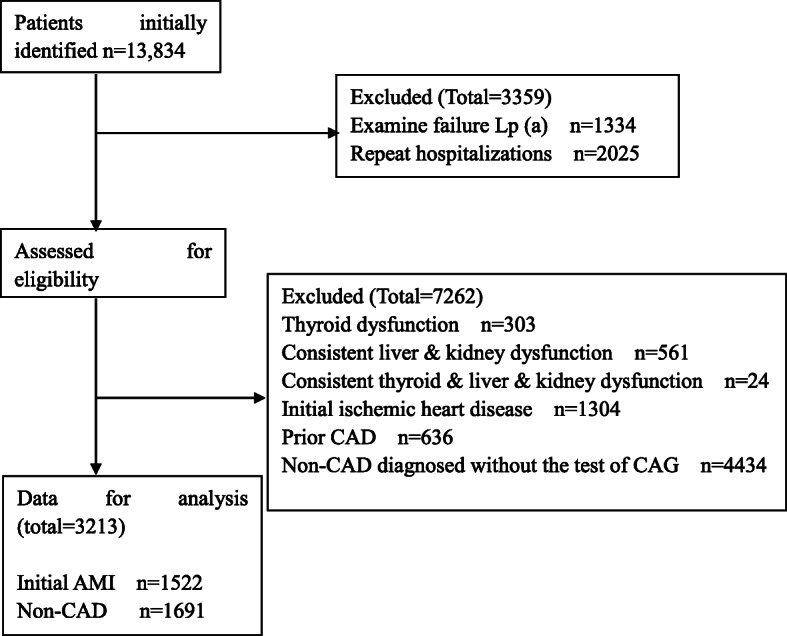
Flow diagram of patient selection. Abbreviations: Lp(a), lipoprotein(a); initial AMI, initial acute myocardial infarction; CAD, coronary artery disease; CAG, coronary angiogram

As described in detail elsewhere [11], a total of 13,834 person-time hospitalized patients were retrospectively included for analysis, among whom, 10,621 were excluded based on the exclusion criteria. As a result, a total of 3213 patients with 1522 cases with initial AMI and 1691 controls without CAD confirmed by coronary angiography met for final analysis. Details were shown in Fig. 1. Baseline characteristics for initial AMI group and non-CAD group were given particularly in Table 1 reprinted from references [12].

**Table 1:** Baseline characteristics

Characteristics	Total	Initial AMI	Non-CAD	P value
N	3213	1522	1691	
Demographic data				
Age (IQR), year	63 (15)	64 (18)	62 (13)	< 0.001
Male n (%)	2117 (65.9)	1229 (80.8)	888 (52.5)	< 0.001
Height (IQR), cm	165 (10)	165 (9)	163 (12)	< 0.001
Weight (IQR), kg	65 (11)	65 (7)	65 (14)	0.693
BMI (IQR)	24.1 (3.0)	24.1 (1.4)	24.4 (4.0)	< 0.001
Marriage n (%)				0.019
Divorced	3 (0.1)	3 (0.2)	0 (0.0)	
Married	3168 (98.6)	1492 (98.0)	1676 (99.1)	
Unmarried	25 (0.8)	15 (1.0)	10 (0.6)	
Widowed	17 (0.5)	12 (0.8)	5 (0.3)	
Life styles				
Smoking status n (%)				< 0.001
Never	1681 (52.3)	578 (38.0)	1103 (65.2)	
Past smoking	276 (8.6)	123 (8.1)	153 (9.1)	
Current smoking	1256 (39.1)	821 (53.9)	435 (25.7)	
Drinking status n (%)				< 0.001
Never	2488 (77.4)	1108 (72.8)	1380 (81.6)	
Past drinking	73 (2.3)	35 (2.3)	38 (2.3)	
Current drinking	652 (20.3)	379 (24.9)	273 (16.1)	
Past history n (%)				
PH	2029 (63.2)	926 (60.8)	1103 (65.2)	0.010
DM	616 (19.2)	346 (22.7)	270 (16.0)	< 0.001
Blood analysis				
Total protein (IQR), g/l	66.4 (8.1)	64.8 (7.7)	67.4 (7.7)	< 0.001
Albumin (IQR), g/l	41.1 (5.5)	39.5 (5.8)	42.1 (4.9)	< 0.001
Creatinine (IQR), μmol/l	74.0 (24.0)	78.0 (24.1)	72.0 (23.0)	< 0.001
ALT (IQR), u/l	26.0 (29.0)	40.2 (39.8)	19.0 (13.0)	< 0.001
AST (IQR), u/l	30.0 (97.0)	123.5 (245.0)	22.0 (8.0)	< 0.001
Hemoglobin (IQR), g/l	135.0 (19.0)	135.0 (20.0)	134.0 (19.0)	0.375
Lipid profiles				
TC, mmol/l	4.1 (1.3)	4.1 (1.3)	4.0 (1.2)	< 0.001
TG, mmol/l	1.3 (1.0)	1.2 (1.0)	1.3 (1.0)	0.009
Lp(a), mg/l	88 (158)	111 (192)	71 (126)	< 0.001
Apo A, g/l	1.3 (0.2)	1.2 (0.2)	1.3 (0.2)	< 0.001
Apo B, g/l	0.9 (0.3)	0.9 (0.3)	0.9 (0.3)	< 0.001
HDL-C, mmol/l	1.1 (0.3)	1.0 (0.3)	1.1 (0.3)	< 0.001
LDL-C, mmol/l	2.5 (1.0)	2.6 (1.1)	2.4 (0.9)	0.038
Medications n (%)				0.001
Rosuvastatin	326 (15.7)	223 (15.9)	103 (15.5)	
Fluvastatin	11 (0.5)	3 (0.2)	8 (1.2)	
Atorvastatin	897 (43.3)	638 (45.4)	259 (39.0)	
Simvastatin	837 (40.4)	542 (38.6)	295 (44.4)	
Imaging				
CAG n (%)	3076 (95.7)	1385 (91.0)	1691 (100.0)	< 0.001

*Note:* Continuous variables were expressed as median (inter quartile range, IQR); categorical variables were expressed as percentage. *Abbreviations: N* number; *CAD* coronary artery disease; *initial AMI* initial acute myocardial infarction; *BMI* body mass index; *PH* primary hypertension; *DM* diabetes mellitus; *ALT* alanine aminotransferase; *AST* aspartate aminotransferase; *TC* total cholesterol; *TG* triglyceride; *Lp(a)*, lipoprotein(a); *Apo A* apolipoprotein A1; *Apo B* apolipoprotein B; *HDL-C* high density lipoprotein cholesterol; *LDL-C* low density lipoprotein cholesterol; *CAG* coronary angiogram

### Odds ratios of first incident AMI in higher BMI alone

Compared with the reference group, OR (95%CI) of first incident AMI was 1.90(1.34–2.69) for higher BMI alone, while after multivariable adjustments, OR (95%CI) was strengthened to 2.27(1.46–3.52). Details were seen in Table 2.

**Table 2:** Odds ratios of first incident AMI for elevated Lp(a) and BMI

Lp(a)	BMI < 24			BMI ≥ 24			^b^ORs(95% CI) for higher BMI within strata of Lp(a)
	N(ca/co)	Crude ^a^OR (95% CI)	^a^OR (95% CI)	N(ca/co)	Crude ^a^OR (95% CI)	^a^OR (95% CI)	
Q1	177/61	1.0	1.0	254/166	1.90 (1.34–2.69)	2.27 (1.46–3.52)	2.27 (1.46–3.52)
Q2	150/82	1.59 (1.07–2.36)	1.79 (1.11–2.90)	204/195	2.77 (1.95–3.94)	2.86 (1.85–4.40)	1.71 (1.14–2.56)
Q3	161/90	1.62 (1.10–2.39)	1.65 (1.05–2.60)	189/212	3.25 (2.29–4.62)	3.30 (2.14–5.11)	1.93 (1.32–2.82)
Q4	137/103	2.18 (1.48–3.21)	1.96 (1.20–3.20)	154/237	4.46 (3.13–6.37)	4.43 (2.76–7.09)	2.56 (1.68–3.91)
Q5	137/137	2.90 (1.99–4.22)	2.34 (1.47–3.71)	128/239	5.42 (3.77–7.79)	5.98 (3.72–9.60)	2.43 (1.63–3.62)
^c^OR(95% CI) for higherLp(a) within strata of BMI			1.79 (1.11,2.90)			1.26 (0.91–1.75)	
			1.65 (1.05,2.60)			1.38 (0.99–1.93)	
			1.96 (1.20,3.20)			1.68 (1.18–2.39)	
			2.34 (1.47–3.71)			2.23 (1.56–3.17)	

*Note:* Q1-Q5: quintile of Lp(a), Q1: ≤34 mg/L, Q2: ≤65 mg/L, Q3: ≤118 mg/L, Q4: ≤246.9 mg/L, Q5: ≤2138 mg/L. a, comparing with BMI < 24 kg/m2 and Lp(a) ≤ 34 mg/L; b, OR of BMI ≥ 24 kg/m2 comparing with BMI < 24 kg/m2 in the same Lp(a) level; c, OR of Lp(a) in Q2-Q5 comparing with Q1 in the same BMI level. Adjustment: age, sex, smoking, drinking, diabetes mellitus, primary hypertension, high density lipoprotein cholesterol, low density lipoprotein cholesterol, triglyceride, albumin, creatinine. *Abbreviations: Lp(a)* lipoprotein(a); *BMI* body mass index; *AMI*;acute myocardial infarction; *OR* odds ratio; *CI* confidence interval; *ca/co* cases/controls

### Odds ratios of first incident AMI in higher Lp(a) alone

Compared with the reference group, ORs(95%CI) for first incident AMI were 1.59(1.07–2.36), 1.62(1.10–2.39), 2.18(1.48–3.21), and 2.9(1.99–4.22) across Lp(a) quintiles with BMI < 24 kg/m^2^. After multivariable adjustments, higher Lp(a) remained significantly associated with first incident AMI. Details were seen in Table 2.

### Odds ratios of first incident AMI in both higher BMI and higher Lp(a) levels

Compared with the reference group, crude ORs (95%CI) for AMI were 2.77(1.95–3.94), 3.25(2.29–4.62), 4.46(3.13–6.37), 5.42(3.77–7.79) and multivariable-adjusted ORs (95%CI) were 2.86(1.85–4.40), 3.30(2.14–5.11), 4.43(2.76–7.09), 5.98(3.72–9.60), respectively, across the Lp(a) quintiles. The risk of AMI accelerated remarkably from Q3 to Q5 in this group. Details were seen in Table 2.

### Interactive effects of both higher BMI and higher Lp(a) levels on the risk of first incident AMI

The RERI was 1.39 (0.26–2.52) in Q4 and 1.62 (0.21–3.03) in Q5 before adjustment. After multivariable adjustment, the RERI was 2.45 (0.36–4.54) in Q5, and its 95% CIs did not include 0, meaning that the estimated joint effect on the additive scale of Lp(a) and BMI together was greater than the sum of the estimated effects of Lp(a) and BMI alone. Therefore, there was a positive interaction on the additive scale. However, the *P* value of product term of BMI and Lp(a) was > 0.05, indicating that the interaction on the multiplicative scale was negative. Details were seen in Table 3.

**Table 3:** Interaction of Lp(a) with BMI on first incident AMI on additive and multiplicative scale

Lp(a)	RERI(95% CI)	*P*	Product term OR(95% CI)	*P*
Q2 (≤65 mg/l)	−0.03(−1.08–1.02)	0.96	0.77 (0.44–1.35)	0.36
Q3 (≤118 mg/l)	0.38(−0.66–1.42)	0.47	0.89 (0.51–1.54)	0.67
Q4 (≤246.9 mg/l)	1.26(−0.19–2.72)	0.09	0.95 (0.54–1.70)	0.88
Q5 (≤2138 mg/l)	2.45 (0.36–4.54)	0.02	0.96 (0.55–1.68)	0.89

*RERI* relative excess risk due to interaction. Adjustment as in Table 2

## Discussion

In this large cross-sectional study, we have found that BMI and Lp(a) are independently relevant to a high risk of the first incident AMI after adjusting for baseline characteristics, lifestyles and laboratory exams. A combination of high BMI and high Lp(a) is in connection with the highest risk of initial AMI. Significant interaction is found between Lp(a) and BMI in initial AMI on additive scale, but not on multiplicative scale (*P* > 0.05).

### BMI and the first incident AMI

In the current study, a higher BMI was associated with a 2.27-fold increased risk of the first incident AMI, consistent with previous studies [20–22]. Therefore, measures taken against adult obesity may be a preventive strategy against AMI.

### Lp(a) and the first incident AMI

Recent studies have strongly supported a causal relationship between circulating Lp(a) levels and coronary artery disease [1, 3–5]. The Lp(a) molecule is composed of LDL/ apoB-100 core and apolipoprotein(a), both of which are related to regulating platelet aggregation, mediating inflammation and inducing vascular remodeling [23]. Thus, Lp(a) can be regarded as one of the causes of atherosclerosis induction and development [24]. From a genetic perspective, rs10455872 and rs3798220, alleles of two single nucleotide polymorphisms (SNPs) in the Lp(a) gene, are proved to be related to high levels of Lp(a) and the risk of CAD [3]. In our study, it was obvious that the risk of AMI increased prominently in high Lp(a) levels alone, especially from Q3 of Lp(a). Our finding underscores the necessity and importance of reducing plasma Lp(a) levels as a preventive strategy for AMI.

### Interaction between BMI and Lp(a) on first incident AMI

According to our study, a combination of high BMI and high Lp(a) was related to the highest risk of first incident AMI. Significant interaction was found between Lp(a) and BMI in initial AMI on additive scale (RERI 2.45(0.36–4.54) in Q5), which meant that the synergy of Lp(a) and BMI was greater than the sum of their respective effects [9]. Some authors have emphasized statistical interaction in the additive model as the basis for assessing biological interaction especially when the additive and multiplicative interactions are in opposite directions [16, 18, 25]. Therefore, a positive additive interaction seems more plausible than a negative multiplicative interaction and deserves more attention. It is well known that acute coronary events are caused by the rupture of atherosclerotic plaques and the blockage of lumen by thrombosis. Lp(a) can inhibit the formation of active plasmin due to its homology with plasminogen [23]. Coincidentally, obesity is related to increased plasminogen activator inhibitor-1 (PAI-1) [21]. Secondly, Lp(a) has been proved to have an impact on platelet activation or aggregation caused by various agonists [23]. Obesity is also associated with increased platelet activation [26]. Moreover, obesity and high levels of Lp(a) are interacting on mediating inflammatory responses. All signs indicate that the co-existence of high levels of Lp(a) and high BMI may have a synergistic effect on plaque rupture and thrombosis. This interaction between BMI and Lp(a) may modify risk of the AMI. If these associations are causal, our findings suggest that interventions to prevent AMI include not only weight control but also Lp(a) lowering.

### Strengths and limitations

This study has the following advantages and disadvantages. To our knowledge, this study is the first to explore additive and multiplicative interactions between Lp(a) and BMI on risk of AMI. Non-CAD controls were identified by coronary angiography, which was a gold standard for the diagnosis of CAD, enabling us to group individuals more accurately. However, Salim Yusuf found that waist-to-hip ratio would be a stronger indicator of myocardial infarction than BMI [27]. Unfortunately, we lacked the data of waist and hip circumference, as well as information on dietary factors that may possibly modify associations between serum lipid and BMI. In addition, the cross-sectional study design is inevitably open to confounders which may exaggerate or diminish the association between exposure and major outcomes. Thus we incorporated possible risk factors into the logistic model fitting to minimize the impact.

## Conclusions

This study demonstrates that BMI and Lp(a) levels are important factors affecting the risk of AMI. Significant interaction is found between Lp(a) and BMI in initial AMI on additive scale in Chinese Han population, indicating that Lp(a) confers greater risk for initial AMI when BMI is elevated. For those whose BMIs are inadequately controlled, Lp(a) lowering may be an option.

## Data Availability

The data that support the findings of this study are available from the First Affiliated Hospital of Soochow University, but restrictions apply to the availability of these data, which were used under license for the current study, and so are not publicly available. Data are however available from the authors upon reasonable request and with permission of the First Affiliated Hospital of Soochow University.

## Abbreviations

Lp(a): Lipoprotein(a)
BMI: Body mass index
AMI: Acute myocardial infarction
CAD: Coronary artery disease
RERI: Relative excess risk due to interaction
OR: Odds ratio
CI: Confidence interval
CCSSSCC: Case collection and scientific research system for clinical cardiology
CAG: Coronary angiography
IHD: Ischemic heart disease
PH: Primary hypertension
DM: Diabetes mellitus
IQR: Inter quartile range
HDL-C: High-density lipoprotein cholesterol
TG: Triglycerides
Alb: Albumin
Cr: Creatinine
LDL-C: Low-density lipoprotein-cholesterol
SNPs: Single nucleotide polymorphisms

## References

[1] Lee SR, Prasad A, Choi YS, Xing C, Clopton P, Witztum JL, Tsimikas S (2017). LPA gene, ethnicity, and cardiovascular events. Circulation.

[2] Dutta P, Courties G, Wei Y, Leuschner F, Gorbatov R, Robbins CS, Iwamoto Y, Thompson B, Carlson AL, Heidt T (2012). Myocardial infarction accelerates atherosclerosis. Nature.

[3] Helgadottir A, Gretarsdottir S, Thorleifsson G, Holm H, Patel RS, Gudnason T, Jones GT, van Rij AM, Eapen DJ, Baas AF (2012). Apolipoprotein(a) genetic sequence variants associated with systemic atherosclerosis and coronary atherosclerotic burden but not with venous thromboembolism. J Am Coll Cardiol.

[4] Saleheen D, Haycock PC, Zhao W, Rasheed A, Taleb A, Imran A, Abbas S, Majeed F, Akhtar S, Qamar N (2017). Apolipoprotein(a) isoform size, lipoprotein(a) concentration, and coronary artery disease: a mendelian randomisation analysis. Lancet Diab Endocrinol.

[5] Wei WQ, Li X, Feng Q, Kubo M, Kullo IJ, Peissig PL, Karlson EW, Jarvik GP, Lee MTM, Shang N (2018). LPA variants are associated with residual cardiovascular risk in patients receiving statins. Circulation.

[6] Wee CC, Girotra S, Weinstein AR, Mittleman MA, Mukamal KJ (2008). The relationship between obesity and atherosclerotic progression and prognosis among patients with coronary artery bypass grafts the effect of aggressive statin therapy. J Am Coll Cardiol.

[7] Yang Q, He YM, Cai DP, Yang XJ, Xu HF (2016). Risk burdens of modifiable risk factors incorporating lipoprotein (a) and low serum albumin concentrations for first incident acute myocardial infarction. Sci Rep.

[8] Liu C, Xu MX, He YM, Zhao X, Du XJ, Yang XJ (2017). Lipoprotein (a) is not significantly associated with type 2 diabetes mellitus: cross-sectional study of 1604 cases and 7983 controls. Acta Diabetol.

[9] Crump C, Sundquist J, Winkleby MA, Sundquist K (2016). Interactive effects of physical fitness and body mass index on the risk of hypertension. JAMA Intern Med.

[10] Knol MJ, VanderWeele TJ (2012). Recommendations for presenting analyses of effect modification and interaction. Int J Epidemiol.

[11] Cai DP, He YM, Yang XJ, Zhao X, Xu HF (2015). Lipoprotein (a) is a risk factor for coronary artery disease in Chinese Han ethnic population modified by some traditional risk factors: A cross-sectional study of 3462 cases and 6125 controls. Clinica Chimica Acta.

[12] Hu Y, Tao JY, Cai DP, He YM (2020). Interaction of lipoprotein(a) with low-density lipoprotein cholesterol on first incident acute myocardial infarction. Clinica Chimica Acta.

[13] Taylor J (2012). Third universal definition of myocardial infarction. Eur Heart J.

[14] Joint committee issued Chinese guideline for the management of dyslipidemia in a: [2016 Chinese guideline for the management of dyslipidemia in adults]. *Zhonghua xin xue guan bing za zhi* 2016, 44(10):833–853. 10.3760/cma.j.issn.0253-3758.2016.10.005 27903370

[15] Moran A, Gu D, Zhao D, Coxson P, Wang YC, Chen CS, Liu J, Cheng J, Bibbins-Domingo K, Shen YM (2010). Future cardiovascular disease in China: markov model and risk factor scenario projections from the coronary heart disease policy model-China. Circ Cardiovasc Qual Outcomes.

[16] Li R, Chambless L (2007). Test for additive interaction in proportional hazards models. Ann Epidemiol.

[17] VanderWeele TJ (2009). Sufficient cause interactions and statistical interactions. Epidemiology.

[18] Andersson T, Alfredsson L, Kallberg H, Zdravkovic S, Ahlbom A (2005). Calculating measures of biological interaction. Eur J Epidemiol.

[19] Hosmer DW, Lemeshow S (1992). Confidence-interval estimation of interaction. Epidemiology.

[20] Zeller M, Steg PG, Ravisy J, Lorgis L, Laurent Y, Sicard P, Janin-Manificat L, Beer JC, Makki H, Lagrost AC (2008). Relation between body mass index, waist circumference, and death after acute myocardial infarction. Circulation.

[21] Wolk R, Berger P, Lennon RJ, Brilakis ES, Somers VK (2003). Body mass index: a risk factor for unstable angina and myocardial infarction in patients with angiographically confirmed coronary artery disease. Circulation.

[22] Morkedal B, Vatten LJ, Romundstad PR, Laugsand LE, Janszky I (2014). Risk of myocardial infarction and heart failure among metabolically healthy but obese individuals: HUNT (Nord-Trondelag health study), Norway. J Am Coll Cardiol.

[23] Riches K, Porter KE (2012). Lipoprotein(a): cellular effects and molecular mechanisms. Cholesterol.

[24] Klesareva EA, Afanas'eva OI, Donskikh VV, Adamova IY, Pokrovskii SN (2016). Characteristics of lipoprotein(a)-containing circulating immune complexes as markers of coronary heart disease. Bull Exp Biol Med.

[25] Ahlbom A, Alfredsson L (2005). Interaction: a word with two meanings creates confusion. Eur J Epidemiol.

[26] Davi G, Guagnano MT, Ciabattoni G, Basili S, Falco A, Marinopiccoli M, Nutini M, Sensi S, Patrono C (2002). Platelet activation in obese women: role of inflammation and oxidant stress. JAMA.

[27] Yusuf S, Hawken S, Ounpuu S, Bautista L, Franzosi MG, Commerford P, Lang CC, Rumboldt Z, Onen CL, Lisheng L (2005). Obesity and the risk of myocardial infarction in 27,000 participants from 52 countries: a case-control study. Lancet.

